# Give It a Tug and Feel It Grow: Extending Body Perception Through the Universal Nature of Illusory Finger Stretching

**DOI:** 10.1177/2041669515599310

**Published:** 2015-10-19

**Authors:** Roger Newport, Kelly Auty, Mark Carey, Katie Greenfield, Ellen M. Howard, Natasha Ratcliffe, Hayley Thair, Kristy Themelis

**Affiliations:** School of Psychology, University of Nottingham, UK; British Psychological Society, St Andrews House, Leicester, UK; School of Psychology, University of Nottingham, UK

**Keywords:** multisensory illusion, finger stretching, children, MIRAGE, not rubber hand illusion

## Abstract

If British teenage boy asks you to pull his finger, it is usually an indication that he simultaneously wishes to break wind. If you were to tell him that you could pull his finger and stretch it to twice its length, you might expect a similarly irreverent response yet when we pulled the fingers of nearly 600 children and adolescents, 93% reported the illusion of stretching. Grossly distorted body representations need not be the preserve of clinical disorders and can reliably be induced in healthy participants across all ages.

## Introduction

Regardless of normal outward appearance, inaccurate and implausible body descriptions are reported in a range of conditions: Those with complex regional pain syndrome, chronic lower back pain, anorexia nervosa, and brain damage can variously describe body parts as being detached, enlarged, missing, duplicated, or simply the wrong shape (Giummarra, Gibson, Georgiou-Karistanis, & Bradshaw, 2008; Keizer et al., 2013; Lewis & McCabe, 2010; Moseley, 2008). The subjective nature of disturbed body representations makes them difficult to understand; only the individuals affected experience them, and their unusual presentations are hard to imagine. Experimentally induced illusions that reliably disturb body perception in healthy individuals are a useful tool for revealing how abnormal representations might be created, become “real,” and be treated (Moseley, Gallace, & Spence, 2011). Understanding how sensory integration influences cortical representations and conscious perceptions of the body, and how that might become aberrant or be manipulated therapeutically, has important clinical implications. Stretched fingers (Preston & Newport, 2011) have been used to explain body representations to over 15,000 people at public engagement events since 2009 (∼1 km of superfluous digitation), yet the universal nature of this illusion has not yet been investigated.

Using a MIRAGE illusion box (www.miragelab.co.uk), 593 children (aged 8–15) were given a two alternative forced choice task: Stating whether it felt like his or her finger had *really* been stretched. MIRAGE, a mediated-reality device, displays live (delay ∼10 ms) video feed of the participant’s own hand in the same spatial plane and location as the real hand (Newport, Pearce, & Preston, 2010). The effect is that of looking through a window at one’s own hand. Participants were randomly selected at a science festival: Some were asked if they would like to have a finger stretched, some were encouraged to “stick your hand in there” while others queued up simply because there was a queue. All consented to take part via registration and were free to withdraw at any time (which some did). Ethics were in accordance with the Declaration of Helsinki.

**Figure 1.**
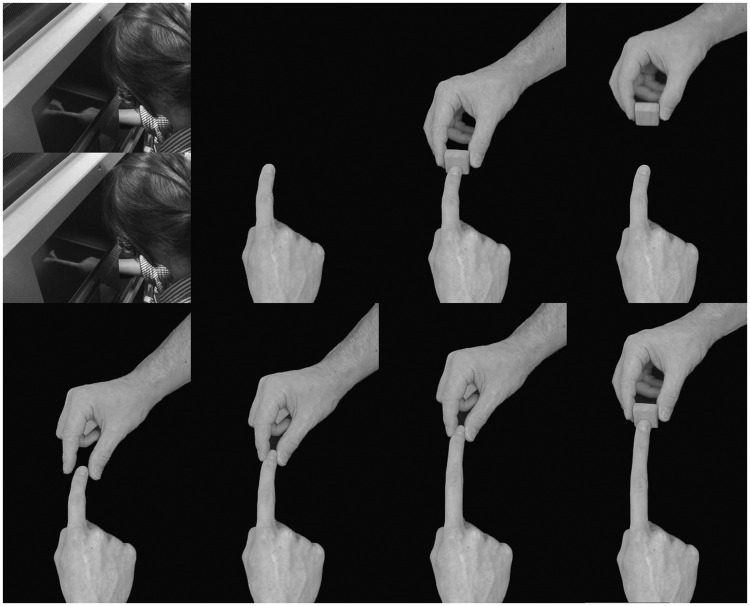
Upon placing his or her hand inside MIRAGE (upper left), each participant was instructed to make a fist and extend the index finger as if pointing at the experimenter (although it is rude to point, it was excused on this occasion). The experimenter placed a wooden block against the fingertip and asked whether the participant could feel it. The block was immediately positioned further away with the words, “But when I move it back here you cannot touch it without moving your hand, can you?” Once agreement had been obtained, the experimenter grasped the distal phalanx (end) of the index finger and gently pulled; firm enough to straighten the collateral ligaments of the finger, but not enough to cause discomfort. Simultaneously, the portion of the live video corresponding to the proximal interphalangeal joint (middle or second knuckle) expanded in such a way that the visible area gradually doubled outwards until it reached the extreme ends of the finger (see Supplemental Video http://www.psychology.nottingham.ac.uk/staff/rwn/Giveitatugsupplementalvideo.mp4). After the stretch, which took 1 to 3 seconds depending upon actual finger length, the experimenter reproduced the wooden block and again touched it against the fingertip with the words, “But now you can!” At this point, the experimenter posed the question, “Does it feel like your finger has *really* stretched?” to which the participant responded “yes” or “no.”

**Figure 2.**
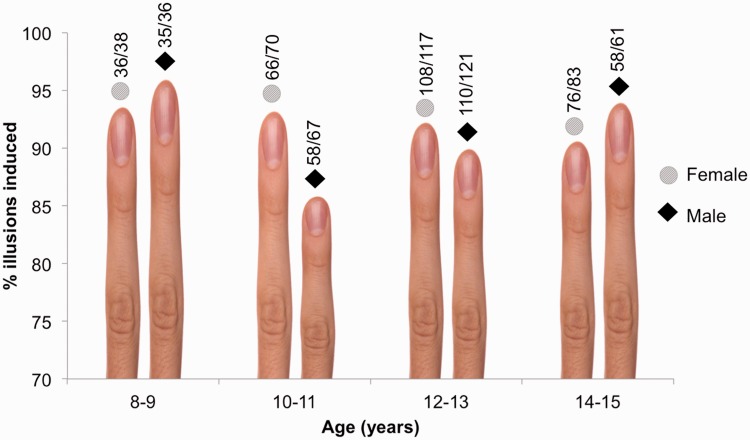
Illusions induced for each age group by gender (females: circles; males: diamonds).

Each participant received a single finger stretch adhering to the protocol outlined in Figure 1. Overall, 93% reported the illusion that their finger had been stretched (Figure 2). Typical reactions were laughter, disgust, amazement, and drooling. The overriding verbal description was that it was “weird” (other descriptions cannot be printed here). For analysis, children were grouped into 2-year bins by gender to investigate two alternative hypothetical outcomes: (a) if sensory integration matures slowly (Assaiante, Barlaam, Cignetti, & Vaugoyeau, 2014), then susceptibility should increase with age; (b) given the skeptical and truculent nature of teenagers (reference your own life), with greater experience of what fingers should or should not do, susceptibility should decrease with age. However, no differences between groups were observed after corrections for multiple comparisons (min: χ^2^(1) = 5.35), supporting neither hypothesis. These results were obtained outside the laboratory, and while some participants were told to expect a stretched finger (something which deserves further investigation), the illusion worked consistently regardless of skepticism, gullibility, prior expectations, positive or negative peer pressure, background noise or sensory load.

From a bottom–up perspective, finger stretching is compelling because it happens to the participant’s own body through simultaneous, congruent manipulation of vision, touch, and proprioception: Apart from elongation, the appearance of the finger is still that of the participant’s own while the felt pull and touch are both congruent with visual elongation. From a top-down perspective, the stretch and the resultant ability to touch a previously distant object are implausible. Many body illusions involve a tussle between bottom-up- and top-down processes; low-level sensory integration informs the brain about body shape, location, or appearance whilst higher level cognitive processes identify discrepancies with knowledge and experience. Importantly, this illusion demonstrates that body perception is not constrained by the physical body or prior experience of the body.

Despite a lifetime with unyielding digits, finger stretching is a powerful and instantaneous demonstration that body perception is a dynamic process. The brain continuously constructs a perception of the body based on the integration of sensory signals that we have little control over, resulting in perceptual experiences that can stretch the boundaries of what we think we know about our own bodies.

## References

[bibr1-2041669515599310] Assaiante C. Barlaam F. Cignetti F. Vaugoyeau M. (2014) Body schema building during childhood and adolescence: A neurosensory approach. Clinical Neurophysiology 44: 3–12. 24502900 10.1016/j.neucli.2013.10.125

[bibr2-2041669515599310] Giummarra M. J. Gibson S. J. Georgiou-Karistanis N. Bradshaw J. L. (2008) Mechanisms underlying embodiment, disembodiment and loss of embodiment. Neuroscience and Behavioural Reviews 32: 143–160. 10.1016/j.neubiorev.2007.07.001 17707508

[bibr3-2041669515599310] Keizer A. Smeets M. A. M. Dijkerman H. C. Uzunbajakau S. A. Van Elburg A. Postma A. (2013) Too fat to fit through the door: First evidence for disturbed body-scaled action in anorexia nervosa during locomotion. PloS One 8: E64602. 23734207 10.1371/journal.pone.0064602 PMC3667140

[bibr4-2041669515599310] Lewis J. McCabe C. (2010) Body perception disturbance (BPD) in CRPS. Practical Pain Management 10: 60–66.

[bibr5-2041669515599310] Moseley G. L. (2008) I can’t find it! Distorted body image and tactile dysfunction in patients with chronic back pain. Pain 140: 239–243. 18786763 10.1016/j.pain.2008.08.001

[bibr6-2041669515599310] Moseley G. L. Gallace A. Spence C. (2011) Bodily illusions in health and disease: Physiological and clinical perspectives and the concept of a cortical “body matrix”. Neuroscience and Behavioural Reviews 36: 34–46. 10.1016/j.neubiorev.2011.03.013 21477616

[bibr7-2041669515599310] Newport R. Pearce R. Preston C. (2010) Fake hands in action: Embodiment and control of supernumerary limbs. Experimental Brain Research 204: 385–395. 20012536 10.1007/s00221-009-2104-y PMC2895889

[bibr8-2041669515599310] Preston C. Newport R. (2011) Analgesic effects of multisensory illusions in osteoarthritis. Rheumatology 50: 2314–2315. 21447568 10.1093/rheumatology/ker104 PMC3222845

